# Changes in the Viral Distribution Pattern after the Appearance of the Novel Influenza A H1N1 (pH1N1) Virus in Influenza-Like Illness Patients in Peru

**DOI:** 10.1371/journal.pone.0011719

**Published:** 2010-07-27

**Authors:** Victor Alberto Laguna-Torres, Jorge Gómez, Patricia V. Aguilar, Julia S. Ampuero, Cesar Munayco, Víctor Ocaña, Juan Pérez, María E. Gamero, Juan Carlos Arrasco, Irmia Paz, Edward Chávez, Rollin Cruz, Jaime Chavez, Silvia Mendocilla, Elizabeth Gomez, Juana Antigoni, Sofía Gonzalez, Cesar Tejada, Gerardo Chowell, Tadeusz J. Kochel, the Peru Influenza working group

**Affiliations:** 1 Virology Department, United States Naval Medical Research Center Detachment, Lima, Perú; 2 Dirección General de Epidemiología, Ministerio de Salud, Lima, Perú; 3 Dirección Regional de Salud de Piura, Ministerio de Salud, Piura, Perú; 4 Universidad Nacional de San Agustín, Arequipa, Perú; 5 Centro Médico Militar 32o Brigada de Infantería del Ejército, Trujillo, Perú; 6 Jefatura de Salud del Ejército del Perú, Lima, Perú; 7 Dirección Regional de Salud del Callao, Gerencia Regional de Salud del Callao, Lima, Perú; 8 Hospital Daniel Alcides Carrión del Callao, Ministerio de Salud, Callao, Perú; 9 Dirección Regional de Salud de Puno, Ministerio de Salud, Puno, Perú; 10 Hospital Nacional Edgardo Rebagliati Martins, EsSalud, Lima, Perú; 11 Centro Médico Naval, Marina de Guerra Del Perú, Callao, Perú; 12 Hospital Santa Rosa de Lima, Ministerio de Salud, Lima, Perú; 13 Mathematical and Computational Modeling Sciences Center, School of Human Evolution and Social Change, Arizona State University, Tempe, Arizona, United States of America; 14 Division of Population Studies, Fogarty International Center, National Institutes of Health, Bethesda, Maryland, United States of America; U.S. Naval Medical Research Center Detachment/Centers for Disease Control, United States of America

## Abstract

**Background:**

We describe the temporal variation in viral agents detected in influenza like illness (ILI) patients before and after the appearance of the ongoing pandemic influenza A (H1N1) (pH1N1) in Peru between 4-January and 13-July 2009.

**Methods:**

At the health centers, one oropharyngeal swab was obtained for viral isolation. From epidemiological week (EW) 1 to 18, at the US Naval Medical Research Center Detachment (NMRCD) in Lima, the specimens were inoculated into four cell lines for virus isolation. In addition, from EW 19 to 28, the specimens were also analyzed by real time-polymerase-chain-reaction (rRT-PCR).

**Results:**

We enrolled 2,872 patients: 1,422 cases before the appearance of the pH1N1 virus, and 1,450 during the pandemic. Non-pH1N1 influenza A virus was the predominant viral strain circulating in Peru through (EW) 18, representing 57.8% of the confirmed cases; however, this predominance shifted to pH1N1 (51.5%) from EW 19–28. During this study period, most of pH1N1 cases were diagnosed in the capital city (Lima) followed by other cities including Cusco and Trujillo. In contrast, novel influenza cases were essentially absent in the tropical rain forest (jungle) cities during our study period. The city of Iquitos (Jungle) had the highest number of influenza B cases and only one pH1N1 case.

**Conclusions:**

The viral distribution in Peru changed upon the introduction of the pH1N1 virus compared to previous months. Although influenza A viruses continue to be the predominant viral pathogen, the pH1N1 virus predominated over the other influenza A viruses.

## Introduction

The influenza virus causes significant morbidity and mortality worldwide [1]. In 1998, a sentinel surveillance system of influenza and other respiratory viruses was established by the Ministry of Health (MoH) of Peru, and in 2006 the surveillance system's coverage was expanded to include additional surveillance sites [2]. Influenza circulation in Peru has been detected throughout the whole year during 2006 to 2008 [3] and within that period a total of 6,308 patients with influenza-like illness (ILI) were enrolled in this passive surveillance study. At least one respiratory virus was isolated from 2,688 (42.6%) of the patients, with etiologies varying by age and geographical region. Influenza A (25%) was the predominant viral respiratory pathogen in the country; however, circulation of influenza B (9.7%) was more commonly detected during epidemiological weeks (EW) 11 and 27 in 2007. In addition, parainfluenza viruses (3.2%), adenovirus (1.8%), respiratory syncytial virus (0.6%), enterovirus (0.5%), herpes virus (HSV; 2.6%) and other viruses (0.1%) were isolated from patient specimens, which collectively contributed to 8.8% of all ILI cases [3].

As a response of the 2009 WHO global influenza pandemic alert, the MoH of Peru intensified surveillance efforts and on May 2009, the first confirmed case of pH1N1 was identified in a Peruvian citizen returning from New York with a respiratory illness. Following this event, the pH1N1 quickly spread throughout the country [4], [5]. In addition, on May 2009, MoH of Peru started an intensive influenza vaccination campaign. During the pandemic, laboratory diagnosis was only conducted by the Instituto Nacional de Salud (INS-MoH) and the US Naval Medical Research Center Detachment (US NMRCD) using the newly described real-time RT-PCR (rRT-PCR) assay [6]. The US NMRCD laboratory provided laboratory support by processing samples from sentinel surveillance sites while INS-MoH focused on processing samples from other sites in Peru.

Here we report a detailed description of the changes in the viral agent distribution pattern from the sentinel surveillance system before and after the appearance of pH1N1 in Peru.

## Methods

### Case definition and study population

An ILI case was defined as any person with a sudden onset of fever (≥38°C) and cough or sore throat fewer than five days in duration, with or without general symptoms such as myalgias, prostration, headache, or malaise. [7]. The study population included every patient with ILI, regardless of age, who sought attention in participating health centers from January 4^th^ to July 13^th^ 2009.

NMRCD recruited patients (outpatient or inpatient) in 38 hospitals and health centers in 14 cities located in 11 provinces. Sites in the other 14 provinces of Peru were covered by INS-MoH. Clinics were located in northern coastal cities (Tumbes, Piura and Trujillo), southern highlands (Arequipa, Cusco and Puno), jungle region (Iquitos, Puerto Maldonado, Junin and Pucallpa) and central coast (Lima) [3]. Specimens from Lima were collected from patients reporting to the Social Security (EsSalud) Hospital and general hospitals maintained by the Peruvian Army, Navy, Air Force, and Ministry of Health. Hospitalization was defined as a patient spending at least one night in the hospital or healthcare center. Both inpatients and outpatients were enrolled.

### Specimens and data collection

Data were collected at the time of medical attention using a case report form (CRF) from all patients who met the case definition criteria. The variables analyzed in our study are shown in Table 1. Viral isolation during this surveillance system was only performed in NMRCD laboratory. Samples included in this report were collected in NMRCD's sites.

**Table 1:** Characteristics of the study population, by period, January–July 2009.

	EW 1 to 18		EW 19 to 28	
CHARACTERISTICS OF STUDY POPULATION	Count	%	Count	%
**Number of patients enrolled**	1422	100.0	1450	100.0
**Respiratory virus positive patients**	505	35.5	730	50.3
**Positive for pH1N1 virus**	0	0.0	395	27.2
**Positive for influenza A non-pH1N1**	**316**	**22.2**	**128**	**8.8**
**Gender** *				
Male	176	55.7	251	48.0
**Age** *				
Mean, ±Std	19.2,±16.5		17.9, ±14.9	
Median, [range]	16, [0,69]		13, [0,87]	
Total	315	22.2	520	35.9
0–4	75	23.8	57	11.0
5–14	72	22.9	237	45.6
15–29	96	30.5	133	25.6
30–44	41	13.0	56	10.8
45–59	21	6.7	28	5.4
> = 60	10	3.2	9	1.7
missing	01		03	
**Regions** *				
Lima	11	3.5	346	66.2
Northern Coast	167	52.8	58	11.1
Southern Highlands	10	3.2	112	21.4
Jungle region	128	40.5	7	1.3
**Travel (within 7 days of symptoms onset)**	30	9.5	50	9.6
**Vaccination history**	5	1.6	62	11.9
**Hospitalized**	2	0.6	50	9.6
**Medical attention before enrollment**	112	35.4	246	47.0
**Previous treatment**				
Antibiotics	44	13.9	117	22.4
Others	4	1.3	22	4.2
No treatment	251	79.4	313	59.8
**Military population**	28	8.9	14	2.7

*From influenza positive patients.

Table 1 shows the total number of patients enrolled in the study, the number of positive cases for at least one virus by isolation or rRT-PCR and describes general characteristics of non-pH1N1 influenza A cases and pH1N1 cases.

One oropharyngeal swab was collected from each patient and stored at −70°C until it could be transported, on dry ice, to Lima by plane or car. At NMRCD in Lima, the specimens were analyzed by rRT-PCR as previously described by the CDC [6] Furthermore, samples were inoculated into four cell lines for virus isolation and identification by immunofluorescence assay (IFA) as previously described [3]. Influenza A virus isolates were defined as non-pH1N1 when the specific rRT-PCR for the novel influenza virus was negative but virus isolation for Influenza A was positive. Further subtyping of these viruses was performed in 111 samples (83 from EW 1 to18 and 28 from EW 19 to 28).

A sample was considered positive for pH1N1 virus when rRT-PCR was positive regardless of the virus isolation result. A co-infection was defined as more than one virus present in cell culture.

Two time periods were assessed in our analysis EW 1–18 (1^st^ period) and EW 19–28 (2^nd^ period). These periods correspond to the time intervals before and soon after the first novel influenza cases appeared in Peru. The clinical-epidemiological forms were entered into a database created in Microsoft (MS) Office Access 2003. Furthermore, the database here analyzed was shared with the Epidemiology Directorate at the Peruvian MoH.

Proportions were compared using a Chi-square test (*X* ^2^). Continuous variables with a normal distribution were compared using the Student's t-test (*t*); otherwise, the Mann-Whitney (*U*) test was applied. P values <0.05 were considered statistically significant. Analyses were conducted using SPSS software version 17.0 (SPSS Inc., Chicago, IL).

### Ethics statement

This surveillance protocol was conducted according to the principles expressed in the Declaration of Helsinki and was approved as less than minimal risk research by the NMRC Institutional Review Board (IRB) and the Peruvian MoH (NMRCD.2002.0019), written consent forms were not required. Stamped, approved information sheets were used in place of written consent forms.

## Results

A total of 2,872 patients were enrolled in this study (Table 1). A total of 1,422 patients were recruited during surveillance activities before the appearance of pH1N1 (EW 1–18), and 1450 patients were recruited during the pandemic period (EW 19–28). Before the appearance of novel virus, non-pH1N1 influenza A virus predominated throughout the country. The age of patients ranged from ≤1 to 87 y with a median age of 16 and 13 y (mean age of 19 and 17.9 y) before and after the identification of the first pH1N1 case in Peru, respectively (*U*, p >0.05).

Until EW 18, a total of 505 (35.5%) patients were positive for respiratory viruses by virus isolation, and from EW 19–28 a total of 730 (50.3%) patients were positive for respiratory viruses by isolation or rRT-PCR (*X* ^2^, p<0.001) (Table 1).

During the 1^st^ period, 62.6% (316/505) patients were positive for non-pH1N1 and in the 2nd period this number decreased to 17.5% (128/730); also in this period 54.1% (395/730) patients were positive for pH1N1 virus. Based on our data (n = 111), the dominant influenza A non-pH1N1 strain prior to week 19 was subtype H1N1 (A/Brisbane/59/07-like) until EW 8 (February 2009), however, by EW 9 (March, 2009) the dominant subtype was H3N2. During the whole 2^nd^ period the influenza A non-pH1N1 dominant subtype was H3N2 (A/Brisbane/10/07-like).

There was a 7-fold increase in seasonal influenza vaccination rates after the appearance of the pandemic virus (*X* ^2^, p<0.001). This increase in vaccination rates was highest among 5–14 year-olds (22 cases) followed by 25–29 year-olds (16 cases) with influenza diagnosis. An increase in patients seeking medical attention was also observed following the appearance of pH1N1 virus (Table 1, 35.4% vs. 47.0%, before and after; *X* ^2^, p<0.001), which correlated with an increase in the number of hospitalizations during the pandemic period (0.6% vs. 9.6%, before and after; *X* ^2^, p<0.001) (Table 1). The most common clinical symptoms reported by patients were fever, cough, malaise, rhinorrhea, and pharyngeal congestion. We did not find a statistically significant difference between the clinical characteristics of patients infected with the pH1N1 virus and those of patients infected with any other respiratory virus including non-pH1N1 influenza A (*X* ^2^, p>0.05); however, patients infected with RSV experienced more wheezing cough than patients infected with other viruses (*X* ^2^, p<0.001).

### Temporal distribution of viral agents

**Figure 1:**
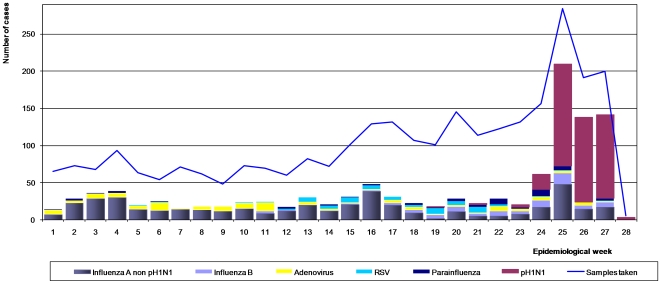
The temporal viral distribution by epidemiological week (EW) in Peru, January 4 to July 13, 2009.

The temporal distribution of viral agents from confirmed cases identified at study sites nationwide is shown in Figure 1.

Before EW 18, non-pH1N1 predominated throughout the country. Although pH1N1 cases began to be detected at the end of EW 18, it was until EW 24 that the number of cases increased considerably and slightly surpassed those of non-pH1N1. Between EW 25 and EW 27, the pH1N1 virus predominated over other viruses (Figure 1).

#### Update information

Figure S1 shows epidemiological data for all weeks, through the end of 2009.

### Viral distribution by region

**Figure 2:**
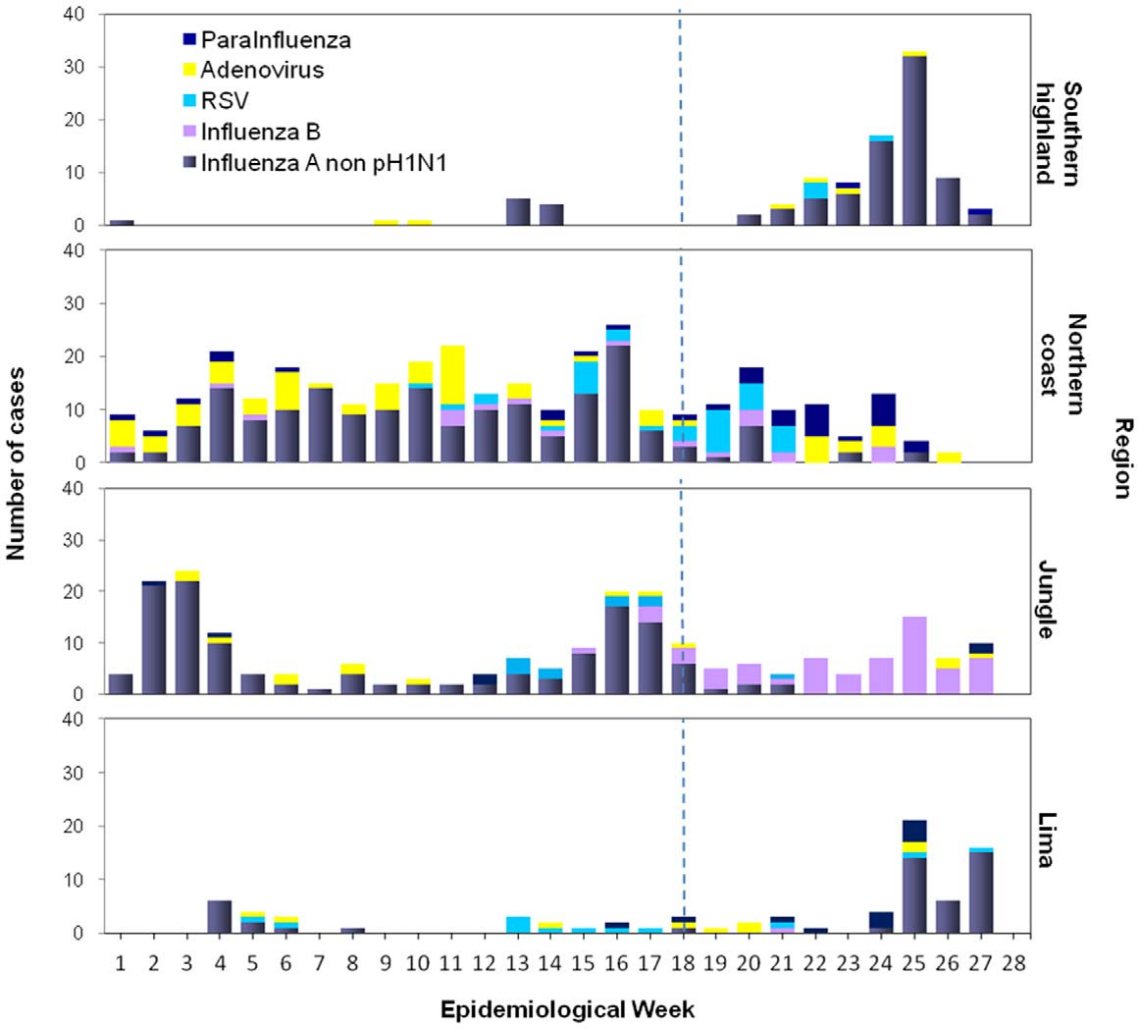
Distribution of viral etiology among regions according to epidemiological week. **Peru, January 4 to July 13, 2009.**

Prior to the appearance of pH1N1, non-pH1N1 and adenovirus were the main viruses isolated regularly from the northern coast. Non-pH1N1 was also the main virus isolated from jungle region. In contrast, few viruses were isolated from patients in Lima and the southern highlands (Figure 2).

**Figure 3:**
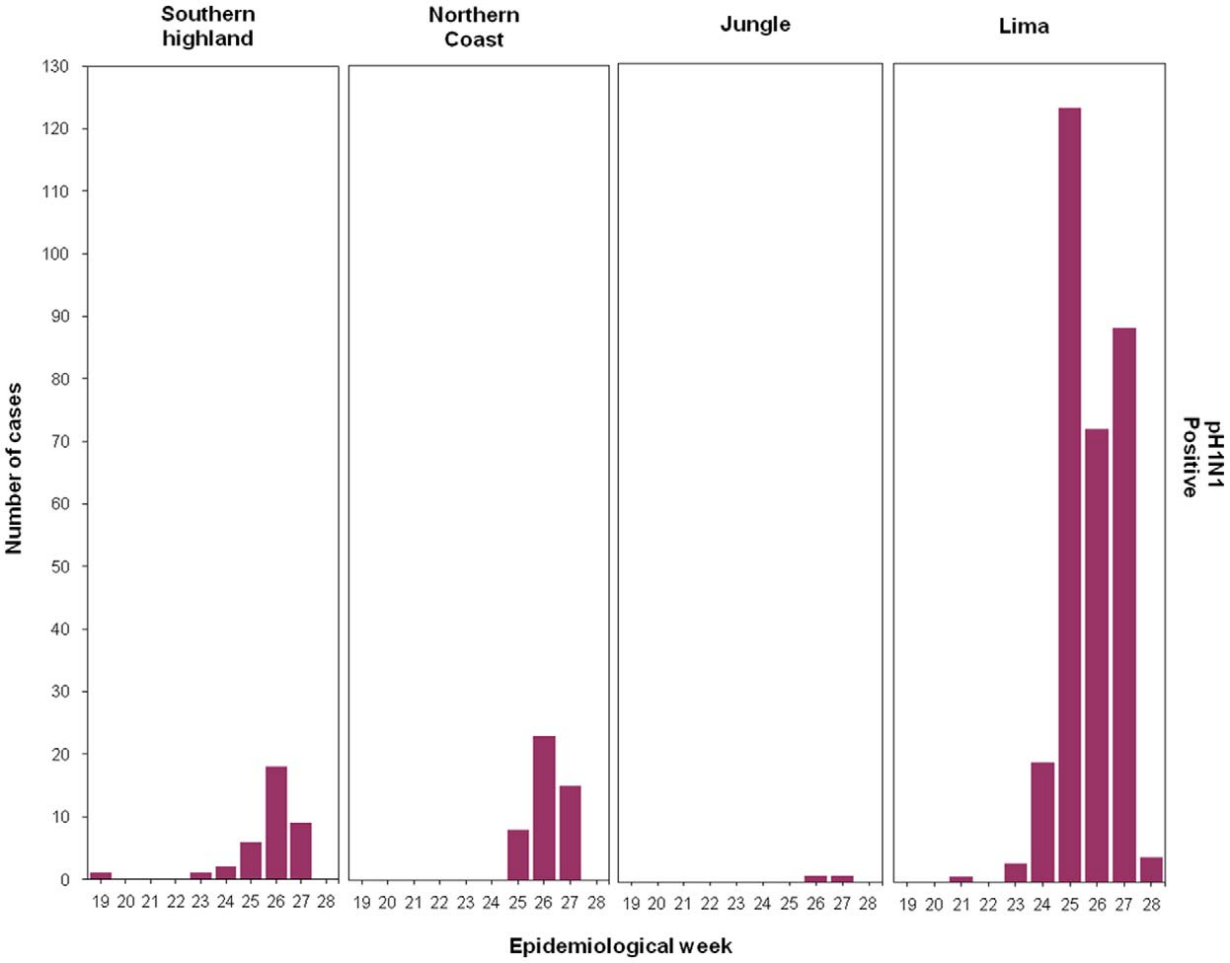
Distribution of Influenza A pH1N1 among regions according to epidemiological week. **Peru, May 10 January to July 13, 2009.**

After EW 18, pH1N1 cases were mainly diagnosed in the capital city of Lima followed by Cusco (southern highlands) and Trujillo (northern coast). In contrast, very few pH1N1 cases were diagnosed in jungle cities (Figure 3). For instance, the city of Iquitos (jungle) experienced the highest number of influenza B cases and only one pH1N1 case (Figure and Table 2).

**Table 2:** Viral etiology of influenza cases by geographical region in Peru, January–July 2009.

				Total			Southern higlands			Northern coast			Jungle region			
				Count		Lima	Arequipa	Cusco	Puno^a^	Tumbes	Piura^b^	Trujillo	Iquitos^c^	Pto. Maldonado	Pucallpa	Junin
**EW 1–18**	**Total**			**1422**	**100.0**	**151**	**17**	**83**	**15**	**193**	**445**	**91**	**311**	**19**	**78**	**19**
	**Positives** *			**505**	**35.5**	**28**	**3**	**13**	**1**	**45**	**215**	**33**	**119**	**9**	**33**	**6**
		Influenza A non-pH1N1		316	**62.6**	11		10		26	110	31	89	8	25	6
			H1N1	48	15.2	3		1		9	8	4	15	3	3	2
			H3N2	35	11.1	5				3	16	5	5		1	
			Non typed	233	73.7	3		9		14	86	22	69	5	21	4
		Influenza B		18	**3.6**					5	6		7			
		HSV		54	**10.7**	2		2	1	5	25	5	13	1		
		RSV		35	**6.9**	9				3	14		4		5	
		Adenovirus		75	**14.9**	4	2			5	52	1	8		3	
		Enterovirus		27	**5.3**	1	1	1			21		2		1	
		Parainfluenza		17	**3.4**	2				1	10		4			
		Others		5	**1.0**						3		2			
	**Negatives**			**917**	**64.5**	**123**	**14**	**70**	**14**	**148**	**230**	**58**	**192**	**10**	**45**	**13**
**EW 19–28**	**Total**			**1450**	**100.0**	**581**	**181**	**70**	**44**	**128**	**181**	**77**	**148**	**16**	**24**	
	**Positives** *			**730**	**50.3**	**376**	**69**	**49**	**14**	**32**	**57**	**51**	**72**	**1**	**9**	
		Influenza A non-pH1N1		128	17.5	36	51	14	10	3	8	1	3		2	
			H1N1	3	2.3	1			1	1						
			H3N2	25	19.5	1	18	1		1	2		1		1	
			Non typed	100	78.1	34	34	13	09	1	6	1	2		1	
		Influenza B		64	8.8	1				6	3		53		1	
		HSV		57	7.8	16	6	3	3	6	10	2	5		6	
		RSV		26	3.6	3	4			4	14		1			
		Adenovirus		25	3.4	5	3	1		3	8	2	3			
		Enterovirus		27	3.7	12	1	1	1	1	5	3	3			
		Parainfluenza		35	4.8	9	1	1		11	11		2			
		Others		10	1.4	2							8			
		pH1N1		395	54.1	310	5	30	2		2	44	1	1		
	**Negatives**			**720**	**49.7**	**205**	**112**	**21**	**30**	**96**	**124**	**26**	**76**	**15**	**15**	

*Denominator for each one of the virus isolated is 505 and 730.

a “Puno” includes data from Puno and Juliaca cities;

b “Piura” includes data from Piura and Sullana cities;

c Iquitos includes data from Iquitos and Yurimaguas cities.

After EW 18, pH1N1 cases were mainly diagnosed in the capital city of Lima and other cities like Cusco and Trujillo. In contrast, jungle cities experienced very few pH1N1 cases. Iquitos (Jungle) exhibited the highest number of influenza B cases and only one pH1N1 influenza A case.

### Viral Etiology before and after the appearance of the pH1N1 virus

**Figure 4:**
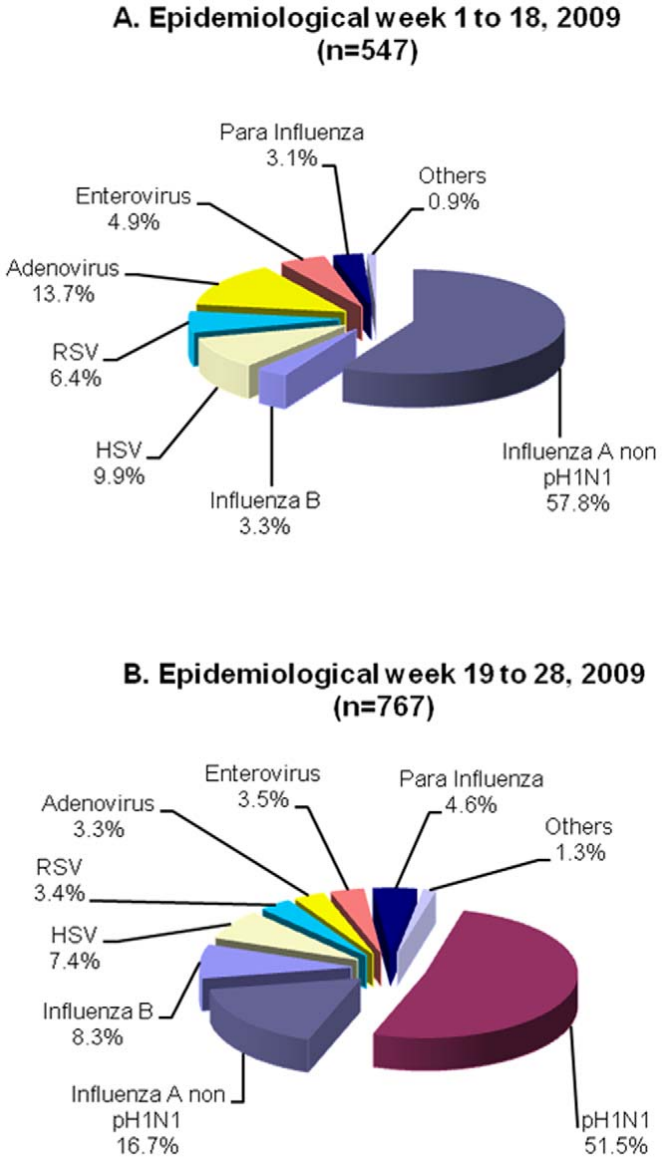
Viral etiology before and after the appearance of the novel pH1N1 virus in Peru. **January 4 to July 13 2009.** A) Before the appearance of the novel pH1N1 virus, a total of 547 positive results were obtained from 505 positive patients; co-infections were found in 42 samples. B) After the appearance of the novel pH1N1 virus, a total of 767 positive results were obtained from 730 positive patients; co-infections were found in 37 samples.

Prior to the appearance of the pH1N1 virus, 547 positive results were obtained from 505 positive patients (42 patients had co-infections). Non-pH1N1 was the predominant viral etiology comprising 57.8% of the confirmed cases (Figure 4A). From EW 19 to 28, the pH1N1 virus was the most prevalent (51.5%) followed by non-pH1N1 (16.7%) (Figure 4B).

### Viral distribution across age groups

**Figure 5:**
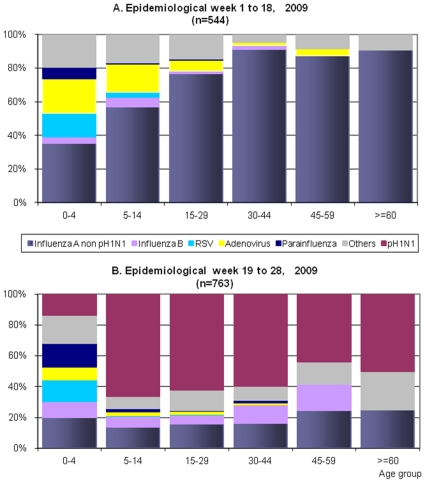
Distribution of viral etiology across age groups before and after the appearance of the novel pH1N1 virus in Peru, January 4 to July 13, 2009. A) Before the appearance of pH1N1 virus a total of 544 positive results were obtained from 505 positive patients (missing = 3). B) After the appearance of the pH1N1 a total of 763 positive results were obtained from 730 positive patients (missing  = 4).

Prior to the appearance of the pH1N1, the most common viral etiologies in all the groups, was non-pH1N1 cases. Figure 5A shows the distribution: non-pH1N1 (35%), adenovirus (20%) and RSV (14%). Influenza B was only detected in 3.8% of cases. The greater 30 years age groups had the highest percentages of non-pH1N1 cases.

After the EW 18, pH1N1 predominated in all age groups except for the infants (0–4 years). In that age group, the non-pH1N1 decreased to 20% (*X* ^2^, p<0.001) and the pH1N1 virus comprised 13.6% of the cases (Figure 5B).

In children 5–14 y, non-pH1N1 decreased from 57.1% to 13.9% after the introduction of the pH1N1 virus. Therefore, pH1N1 influenza cases comprised 66% of the cases. This pattern was also observed among 15–59 year olds. (Figure 5A and 5B).

After EW 18 an increased number of influenza B cases were found among those 30–59 y (Figure 5B). These cases were mostly located in Iquitos (jungle region) where only one case of pH1N1 was diagnosed (Figure and Table 2).

Half of the patients with pH1N1 (n = 196) were between 5–14 years old and 106 (27%) were between 15–29 years of age.

### Co-infections

Before the appearance of the pH1N1 a total of 42 (2.9%) samples had co-infections by isolation. The most frequent co-infections identified were: adenovirus with enterovirus (11 samples), non-pH1N1 influenza A and HSV (11 samples), and non-pH1N1 influenza A and adenovirus (4 samples). After the appearance of the novel virus, 36 (2.50%) samples had co-infections. In nine pH1N1-positive samples viral co-infections were observed, including 7 co-infections with HSV, 1 with an enterovirus, and 1 with a parainfluenza virus. Viral co-infections were observed for seven non-pH1N1 influenza-positive samples, including 3 co-infections with an enterovirus, 3 with HSV, and 1 with RSV. Frequent HSV-enterovirus co-infections (n = 5) were also identified.

## Discussion

Prior to the introduction of this novel influenza virus, our surveillance activities (2006–2008) identified Influenza A as the predominant viral etiology among ILI patients in Peru [3]. In 2009, based on our data, the dominant influenza A non-pH1N1 strain prior to EW 19 was subtype H1N1; this subtype predominated over subtype H3N2 from January through February 2009. However, by March the dominant subtype was H3N2. Interestingly, upon the introduction of the pH1N1, the viral distribution pattern changed compared to the non-pandemic period. Even though influenza A continued to be the predominant viral pathogen, non-pH1N1 influenza A viruses were also rapidly displaced from their predominance by pH1N1. Our data tend to support higher transmission potential of pH1N1 than non-pH1N1 influenza A viruses, as reported elsewhere [4], [8], [9]. This phenomenon could be partially explained by higher population susceptibility to pH1N1 than non-pH1N1 virus to which the population might have acquired partial immunity in previous years via prior natural exposure with antigenically-related strains and annual immunization campaigns of a fraction of the high-risk population, with live attenuated vaccines [10], [11], [12]. A well-known result in theoretical epidemiology is that in a population challenged by multiple infectious agents, the one with the highest reproduction number (fitness) will dominate the transmission dynamics [13]. The reproduction number implicitly accounts for the intrinsic virus transmissibility and the background population immunity. In an earlier report [4], [8], [9] we estimated the reproduction number from the initial pandemic growth phase in the range 1.2–1.7, which is in good agreement with estimates of the reproduction number of inter-pandemic influenza in temperate countries [10].

During the study period, Lima and the southern highlands (Cusco) experienced a more profound impact from the pandemic than other Peruvian regions, going from less than ten cases of ILI per week before, to more than ten cases per week after the pandemic onset. Prior to the appearance of the pH1N1 virus, the majority of these cases were caused by non-pH1N1 influenza A and adenoviruses. The more tropical jungle and northern coastal regions with regular viral isolation did not experience a similar increase in ILI cases, although these geographical regions noticed a change in distribution of detected viruses, from EWs 19–28.

Our data show that influenza positive cases were more frequent among children under 14 y before and after the appearance of the new virus and confirm that the pH1N1 virus in Peru was more frequent among 5–14 y ILI patients [4], which is in agreement with reports from Chile [14]. In other South American countries like Chile, circulation of pH1N1 was first detected after EW 20 (May 17, 2009) and before that week RSV, parainfluenza viruses, and adenoviruses were the predominant viral etiologies among patients with ILI. Moreover increased circulation of the novel influenza virus reached up to 64% of viral isolations at EW27 [15].

In our data, the lower frequency of pH1N1 viral cases among >60 y suggests relative protection for persons who were exposed to H1N1 strains during childhood prior to the 1957 pandemic [16]. However, the number of cases of non-pH1N1influenza A was also low in the same group possibly due to the vaccination campaign initiated by the MoH, complicating the interpretation of our data.

One shortcoming of a sentinel surveillance program is the potential for sampling and selection bias. Therefore our results may not be representative of the entire population of Peru. Hence, this potential bias and the lack of reliable population data preclude us from calculating incidence rates [3]. However, one advantage provided by sentinel surveillance systems is the ability to identify increasing trends in the number of patients seeking medical attention due to ILI symptoms and identifying the viruses related to such increases using fewer resources than a population-based study require.

We found significant changes in hospitalization rates following the introduction of the pandemic virus. This maybe explained by increased awareness among the population about the presence of the new virus in addition to the global alarm on pandemic risk. Of note we did not focus in the long-term follow-up of patients to determine post-enrollment complications and hospitalization rates.

Reports of seasonal influenza vaccination rates increased 7-fold following the appearance of the pH1N1 influenza virus. This increase in vaccination rates could explain the drop in non-pH1N1 influenza A. However, the seasonal influenza vaccination policy in Peru was focused on individuals under 2 y and over 60 y. Hence, the drop in the number of non-pH1N1influenza A cases was most likely driven by strain competition dynamics with the pH1N1, and not by vaccine intervention.

Influenza-like illness circulation will continue to be monitored throughout Peru, to determine when the novel influenza virus reaches non-epidemic levels and detect any further changes in the viral distribution pattern particularly on the fast approaching winter season.

In the pandemic, the viral distribution in Peru changed upon the introduction of the pH1N1 virus compared to previous months. Although influenza A viruses continue to be the predominant viral pathogen, the pH1N1 virus predominated over the other influenza A viruses.

## Supporting Information

Figure S1 Updated results, 2009. Epidemiological week updated information for the whole year. Peru, January 4 to December 31, 2009.
(0.93 MB TIF)
