# Supplementary figures and images for: Changes in the Viral Distribution Pattern after the Appearance of the Novel Influenza A H1N1 (pH1N1) Virus in Influenza-Like Illness Patients in Peru
Source: PLoS One. 2010 Jul 27;5(7):e11719. doi: 10.1371/journal.pone.0011719 (PMC2910706; doi:10.1371/journal.pone.0011719)

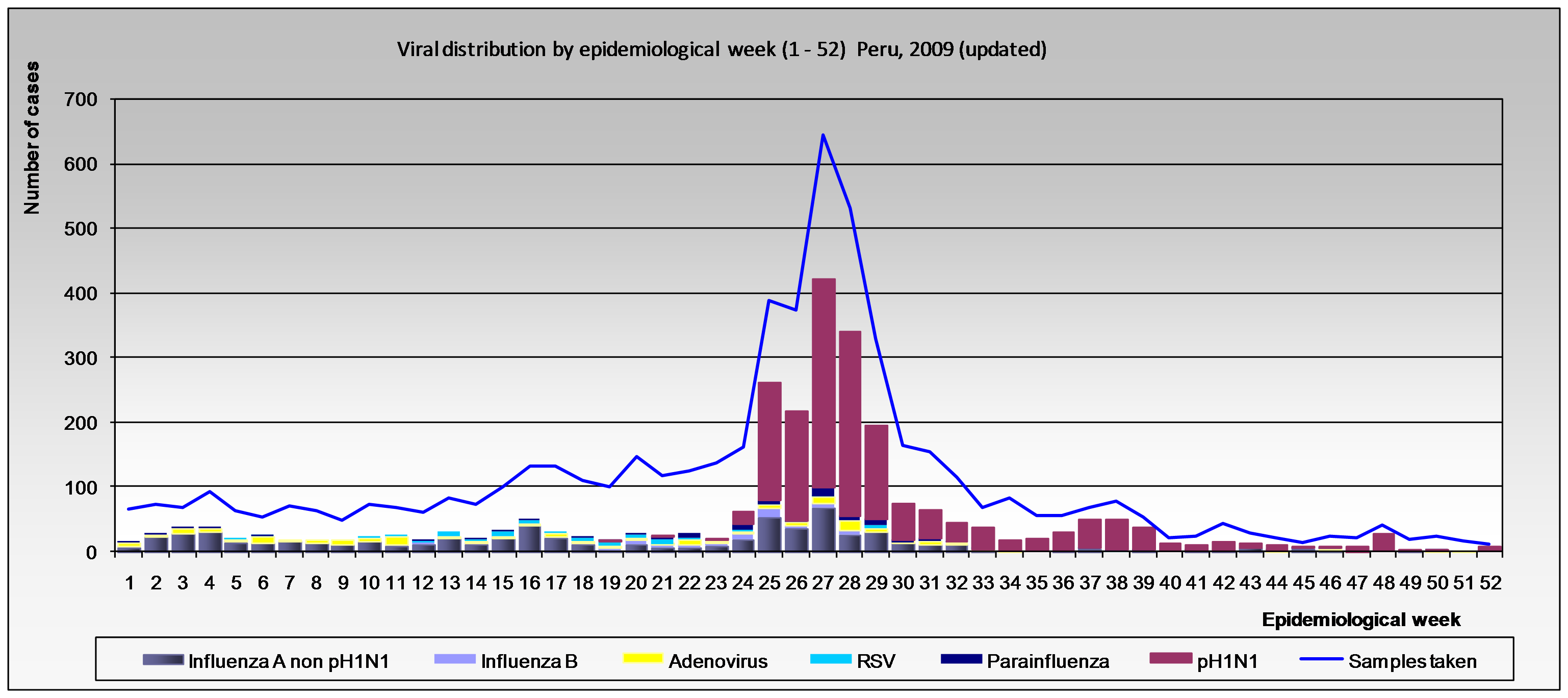

Supplement: Figure S1 — Updated results, 2009. Epidemiological week updated information for the whole year. Peru, January 4 to December 31, 2009. (0.93 MB TIF) [file pone.0011719.s001.tif]
